# The Joint Center for Structural Genomics: exploration of the human gut microbiome

**DOI:** 10.1186/gb-2011-12-s1-i18

**Published:** 2011-09-19

**Authors:** Ian Wilson

**Affiliations:** 1 The Scripps Research Institute, 10550 North Torrey Pines Road, La Jolla, CA 92037, USA

## 

For more than a decade, the Joint Center for Structural Genomics (JCSG) [1] has been at the forefront of developing tools and methodologies that allow the application of high-throughput structural biology to a broad range of biological and biomedical investigations. In the previous phases of the National Institutes of Health’s Protein Structure Initiative (PSI; 2000 to 2010) [2], we explored structural coverage of uncharted regions of the protein universe [3], as well as a single organism, allowing complete structural reconstruction of the metabolic network of *Thermotoga maritima*[*4*]. In the current phase (PSI: Biology; 2010 to 2015), the JCSG is leveraging its high-throughput platform to explore the structural basis for host-microbe interactions in the human microbiome. The emerging field of metagenomics has been particularly enlightening: the human gut microbiome sequencing projects have already uncovered fascinating new families and expansions of known families for adaptation to this environment. The gut microbiota is dominated by poorly characterized bacterial phyla, which contain an unusually high number of uncharacterized proteins that are largely unstudied. Their influence upon human development, physiology, immunity and nutrition is only starting to surface and is thus an exciting new frontier for structural genomics, where we can structurally investigate the contributions of these microorganisms to human health and disease. The JCSG is located at The Scripps Research Institute, the Genomics Institute of the Novartis Research Foundation, University of California at San Diego, the Sanford-Burnham Medical Research Institute and SSRL/Stanford University.

## References

[B1] The Joint Center for Structural Genomics http://www.jcsg.org

[B2] The Protein Structure Initiative http://www.nigms.nih.gov/initiatives/psi

[B3] Jaroszewski L Li Z Krishna SS Bakolitsa C Wooley J Deacon AM Wilson IA Godzik A Exploration of uncharted regions of the protein universe PLoS Biol 2009 7 e1000205 10.1371/journal.pbio.1000205 19787035 PMC2744874

[B4] Zhang Y Thiele I Weekes D Li Z Jaroszewski L Ginalski K Deacon AM Wooley J Lesley SA Wilson IA Palsson B Osterman A Godzik A Three-dimensional structural view of the central metabolic network of *Thermotoga maritima* Science 2009 325 1544 1549 10.1126/science.1174671 19762644 PMC2833182

